# Safety assessment of graphene oxide and microcystin-LR complex: a toxicological scenario beyond physical mixture

**DOI:** 10.1186/s12989-022-00466-x

**Published:** 2022-04-07

**Authors:** Ying Ma, Xiaomeng Ding, Qing Liu, Yanting Pang, Yuna Cao, Ting Zhang

**Affiliations:** Key Laboratory of Environmental Medicine Engineering, Ministry of Education, School of Public Health, Southeast University, Nanjing, 210009 China

**Keywords:** Graphene oxide, Microcystin-LR, Adsorption, Programmed cell death, Toxicity mechanism

## Abstract

**Background:**

Nanomaterials have been widely used in electrochemistry, sensors, medicine among others applications, causing its inevitable environmental exposure. A raising question is the “carrier” effect due to unique surface properties of nanomaterials, which may collectively impact the bioavailability, toxicokinetic, distribution and biological effects of classic toxicants. Noteworthy, this aspect of information remains largely unexplored.

**Methods:**

Here, we deliberately selected two entities to mimic this scenario. One is graphene oxide (GO), which is made in ton quantity with huge surface-area that provides hydrophilicity and π–π interaction to certain chemicals of unique structures. The other is Microcystin-LR (MCLR), a representative double-bond rich liver-toxic endotoxin widely distributed in aquatic-system. Firstly, the adsorption of GO and MCLR after meeting under environmental conditions was explored, and then we focused on the toxicological effect and related mechanism of GO-MCLR complex on human skin cutin forming cells (HaCaT cells) and normal liver cells (L02 cells).

**Results:**

Abiotically, our study demonstrated that GO could effectively adsorb MCLR through hydrogen bonding and π–π interaction, the oxidation degree of GO-MCLR decreased significantly and surface defect level raised. Compared to GO or MCLR, GO-MCLR was found to induce more remarkable apoptosis and ferroptosis in both HaCaT and L02 cells. The underlying mechanism was that GO-MCLR induced stronger intracellular reactive oxygen species (ROS) and mtROS generation, followed by Fe^2+^ accumulation, mitochondrial dysfunction and cytoskeletal damage.

**Conclusions:**

These results suggest that the GO-MCLR complex formed by GO adsorption of MCLR may exhibit more toxic effects than the single material, which demonstrates the necessity for assessing nano-toxicant complexity. Our discovery may serve as a new toxicological paradigm in which nanomaterial mediated surface adsorption effects could impact the degree of cytotoxicity and toxicological mechanisms of classic toxins.

**Graphical Abstract:**

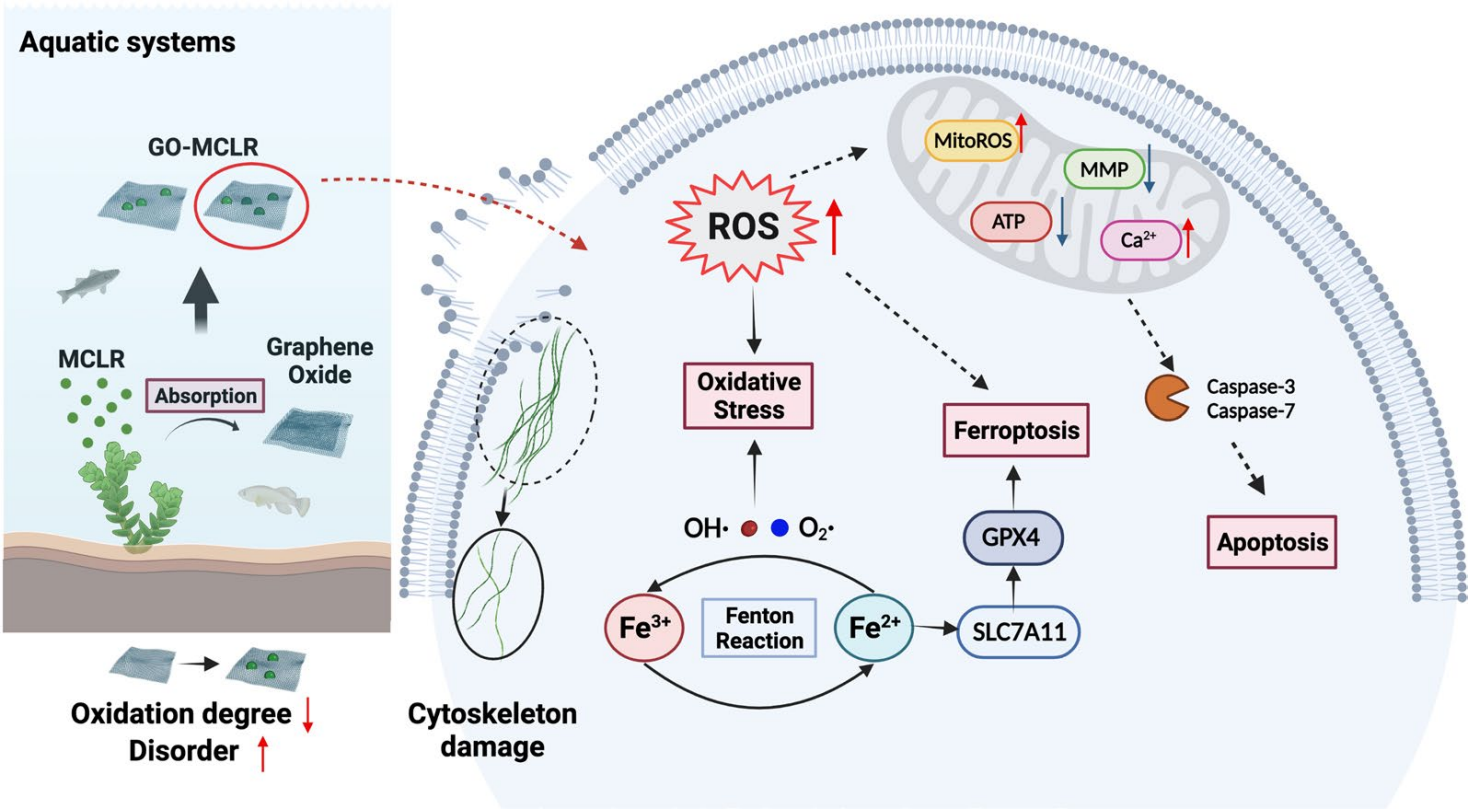

**Supplementary Information:**

The online version contains supplementary material available at 10.1186/s12989-022-00466-x.

## Introduction

Graphene is a single layer of carbon hexagons with sp^2^ hybridized C–C bonding with π-electron clouds [1]. Based on unique physicochemical properties such as large specific surface area, mechanical flexibility, thermal and chemical stability, graphene-based materials are now widely used in commercial production [2–4]. Currently, annual production of graphene has exceeded 15 tons and is expected to reach 3800 tons by 2027, with total annual sales of $300 million [5]. With the rapid growth of production capacity and applications, although there is no clear literature on the amount of graphene in the environment [6], graphene-based materials will inevitably be leaked into the environment during production, storage, transportation, use, disposal and recycling. For example, a large number of graphene releases may occur when it be used in environmental protection, such as adsorption treatment of wastewater and drinking water, solid phase extraction, seawater desalination and coating of filtration materials [7–9]. Due to its superior adsorption performance, graphene is easy to interact with environmental pollutants after being released into the environment, and adsorbents are mainly absorbed on the sp^2^ hybrid carbon atoms of graphene by van der Waals forces. The oxygen-containing functional groups endow graphene oxide (GO) higher affinity for various pollutants, making it gradually replace traditional carbon materials to become a hot spot in water/wastewater treatment. Madadrang et al*.* reported that GO, GO-Ethylene Diamine Tetraacetic Acid (EDTA) and RGO-EDTA formed complexes with Pb^2+^ through surface interaction to remove excess Pb from water [10]. Besides heavy metals, GO has also been proved to be able to effectively adsorb organic compounds in water, including polycyclic aromatic hydrocarbons [11], halogenated aliphatic hydrocarbons [12], antibiotics [13] and hormones [14]. The interaction between GO and other pollutants in the environment is likely to alter the environmental behavior of both itself and the pollutants, as well as their toxicity to organisms.

The main harm caused by cyanobacteria blooms lies in the release of various types of cyanobacteria toxins into water after the bursting of cyanobacteria cells. Among all the different cyanobacteria toxins discovered, Microcystins (MCs) are the cyanobacteria toxins with the highest frequency, the largest amount of production and the most serious harm [15]. In general, the level of microcystin in drinking water is limited to 1 μg/L, and the level of microcystin in normal water is also within this limit. But the MCs content in water varies with time and place. Vezie et al*.* sampled cyanobacteria blooms at five sites in Lake Grand-Lieu on seven different occasions and detected the content of MCs in the field samples, and found that the concentration of MCs in the blooms was up to 230ug/g [16]. The above case indicates that during the special period of cyanobacteria outbreak, the presence of a large number of MCs in water is likely to cause serious environmental and biosafety problems.

MCs are cyclic polypeptides composed of 7 amino acids with a molecular weight of about 1000, also known as monocyclic heptapeptide liver toxin [17]. Due to the structural diversity of MCs caused by changes in amino acid types and positions, more than 200 different types of MCs have been identified. Among them MCLR, MCRR and MCYR (L, R and Y represent leucine, arginine and tyrosine respectively) are the most important three types. MCLR with a molecular weight of about 995.2 g/mol is a powerful tumor promoter, and epidemiological studies in Guangxi and Fujian provinces of China have shown that MCLR is one of the risk factors for high incidence of primary liver cancer [18, 19]. MCLR exerts cytotoxic effects mainly by binding to serine/threonine protein phosphatase 1 (PP1) and protein phosphokinase 2A (PP2A). Then inhibition of PP1 and PP2A activity leads to hyperphosphorylation of various proteins, destruction of hepatocyte skeleton, alteration of cell membrane permeability, and ultimately deformation of hepatocytes [20, 21]. For example, the group of Ma found that MCLR at 5 and 10 μM/L increased phosphorylation of insulin receptor substrate 1, AKT-Ser473, and S6K1-Thr389 by inhibiting the activity of PP2A in in human liver cells [22].

By reviewing the environmental exposure risks of graphene materials and MCLR, it can be found that the two are most likely to coexist in aquatic environments. Currently, GO has been used as an MCLR adsorbent, and in most cases, GO will be reused until its adsorption saturation is reached [23]. It has been reported that nanomaterials can alleviate the risk of co-pollutants by absorbing pollutants and down-regulating their free concentration, but if nanomaterials with pollutants are absorbed by organisms, the toxicity may be enhanced [24]. However, studies have shown that cell membrane rupture caused by sharp edges is one of the main mechanisms of GO cytotoxicity, but such damage could be reduced via the formation of protein crown [25]. As both a polypeptide protein and a classical pollutant, will MCLR reduce its toxicity by forming the protein crown around the sharp edges of GO, or will the two act in synergy? Also, from the point of view of programmed cell death, the manner in which the GO-MCLR polymer leads to cell death has not been identified. This series of scientific problems have not been solved yet, and it is urgent to carry out relevant research. Based on this, this study first explored the adsorption of GO and MCLR after meeting under environmental conditions. Given actual contact and material target organs, the toxicological effect and related mechanism of GO-MCLR complex on human skin cutin forming cells (HaCaT cells) and normal liver cells (L02 cells) were investigated, of which the programmed cell death were focused. By exploring the adsorption effect of GO on MCLR and the toxic effect of complex GO-MCLR, this study aims to elucidate the structural differences between the GO-MCLR complex and the pristine GO, as well as the resulting differences in the toxic effects of the two, highlighting the impact of carrier effects on the environmental health risks of typical nanomaterials and providing principled evidence for the complexity of nano-toxicant safety evaluation.

## Results

### Adsorption and physical–chemical characterizations of GO/GO-MCLR

**Fig. 1:**
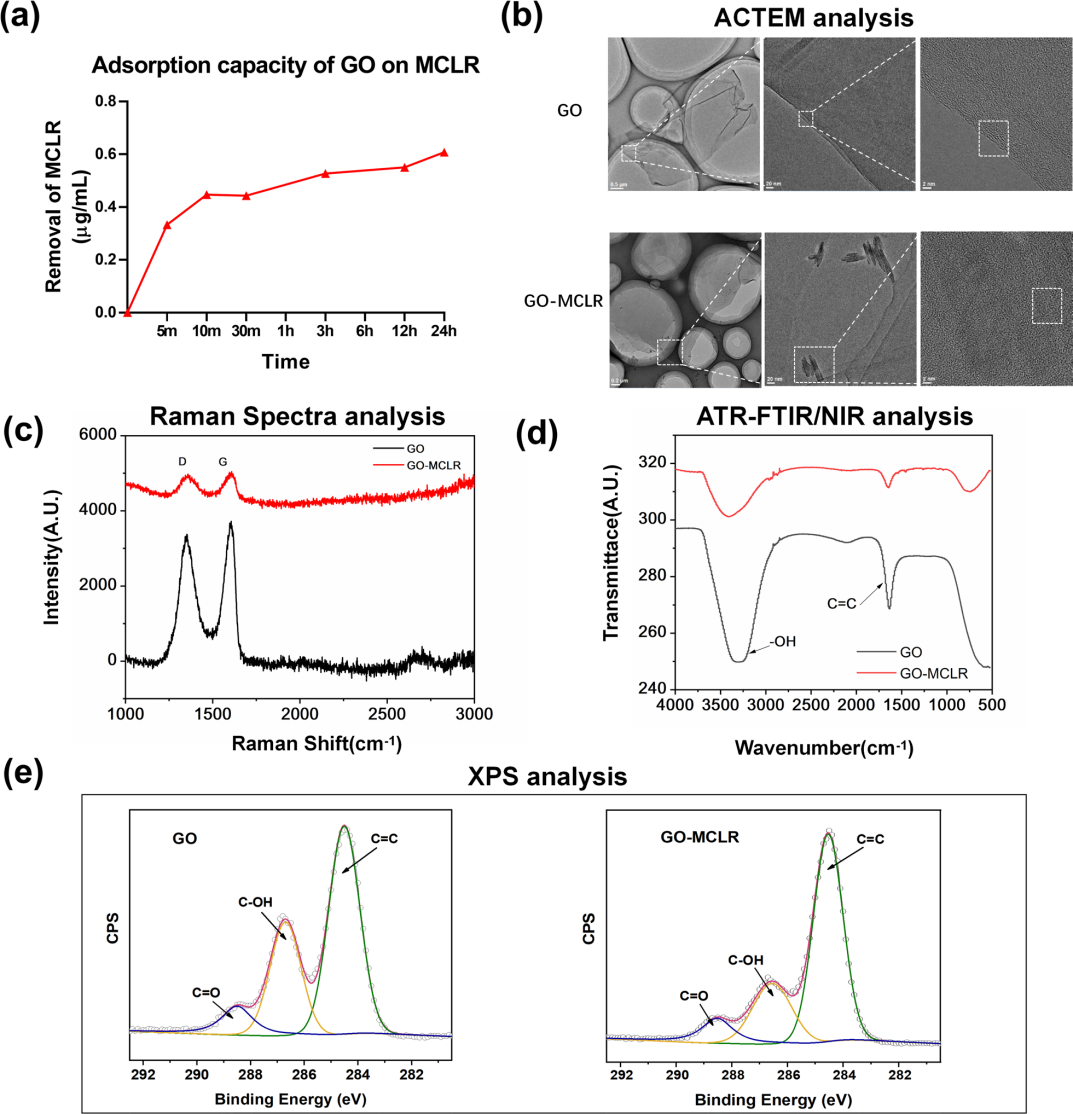
Adsorption of GO on MCLR physical–chemical characterizations of GO/GO-MCLR. A mixed solution of GO-MCLR (50 μg/mL GO + 1 μg/mL MCLR, pH = 7) was prepared by using the GO and MCLR mother liquors with a concentration of 1 mg/mL. **a** The adsorption capacity of GO on MCLR after 5 min, 10 min, 30 min, 3 h, 12 h and 24 h were determined. For the pristine GO and the GO-MCLR composite solution when adsorption equilibrium, **b** TEM analysis of morphology of GO and GO-MCLR; **c** Raman Spectra analysis of structural defects; **d** ATR-FTIR/NIR analysis of GO and GO-MCLR functional groups; **e** XPS quantitative analysis of surface functional groups

In order to evaluate the impact of MCLR adsorption on the bio-hazardous potential of GO, GO-MCLR composite materials were first prepared through adsorption assay which have mentioned before. Figure 1a shows the adsorption of MCLR by GO at different time points in an aqueous solution with pH 7. It can be seen that the adsorption speed of MCLR on GO is relatively fast in the first 30 min, and then adsorption proceeds at a slower speed until adsorption equilibrium about 3 h later. When the adsorption experiment lasted for 24 h, the concentration of MCLR in supernatant decreased by 0.607 μg/mL compared with the initial concentration. According to the adsorption capacity of GO to MCLR when adsorption equilibrium, the GO-MCLR composite solution used in the subsequent experiments was obtained by adding 600 μg MCLR to 1 L GO solution at the concentration of 50 mg/L, then rotating in the dark to ensure full absorption.

The physical and chemical characterization of GO materials before and after adsorption was carried out in detail. The Malvern particle size analyzer showed that the hydrated particle sizes of GO and GO-MCLR in pure water respectively were 14.69 nm and 540.7 nm. The larger size of GO-MCLR compared to GO indicates that MCLR successfully adheres to the GO surface. The microstructures of GO and GO-MCLR were demonstrated by transmission electron microscopy (TEM) images. Figure 1b reveals the surface wrinkles and edge defect structure of GO visible to the naked eye. After magnifying the images, the layer-spacing structure of GO can be observed, which also indicates that GO in this experiment is material with a few layer-spacing structure. From the ACTEM image of GO-MCLR, it can be noticed that MCLR is fusiform and attached to the surface wrinkles of GO. The overlap of MCLR lattice structure and GO honeycomb structure proves that MCLR is successfully adsorbed on GO surface. The Raman spectra of GO and GO-MCLR are shown in Fig. 1c, D and G bands at 1352 cm^−1^ and 1618 cm^−1^ respectively both are the typical peaks indicating graphene, and the intensity ratio of D band to G band (ID/IG) provides an overall indication of the level of GO surface defects. After GO adsorbed MCLR, ID/IG increased from 0.88 to 0.97, indicating that the graphitization of GO-MCLR decreased and disorder degree increased. The Fourier Transform Infrared Spectrometer (FTIR) spectra of GO and GO-MCLR, which help identify functional groups, are shown in Fig. 1d. There are two characteristic peaks identified by the FTIR spectrum of GO, one is the strong and wide peak at 3300 cm^−1^ caused by the stretching vibration of O–H (carboxyl group, hydroxyl group and adsorbed H_2_O), and the other is that the peak at 1650 cm^−1^ due to the tensile vibration of C=C [26]. whereas the strength of the characteristic peak of O–H tensile vibration decreased significantly after GO adsorbed MCLR. In addition, X-ray photoelectron spectroscopy (XPS) was used to characterize the surface functional groups of GO and GO-MCLR, and it was found that the adsorption of GO to MCLR was accompanied by a significant decrease in C=OH (from 32.54% to 28.87%) and C=O (from 22.68% to 21.83%), which shown in Fig. 1e.

### Cytotoxicity induced by GO, MCLR and GO-MCLR in HaCaT cells and L02 cells

**Fig. 2:**
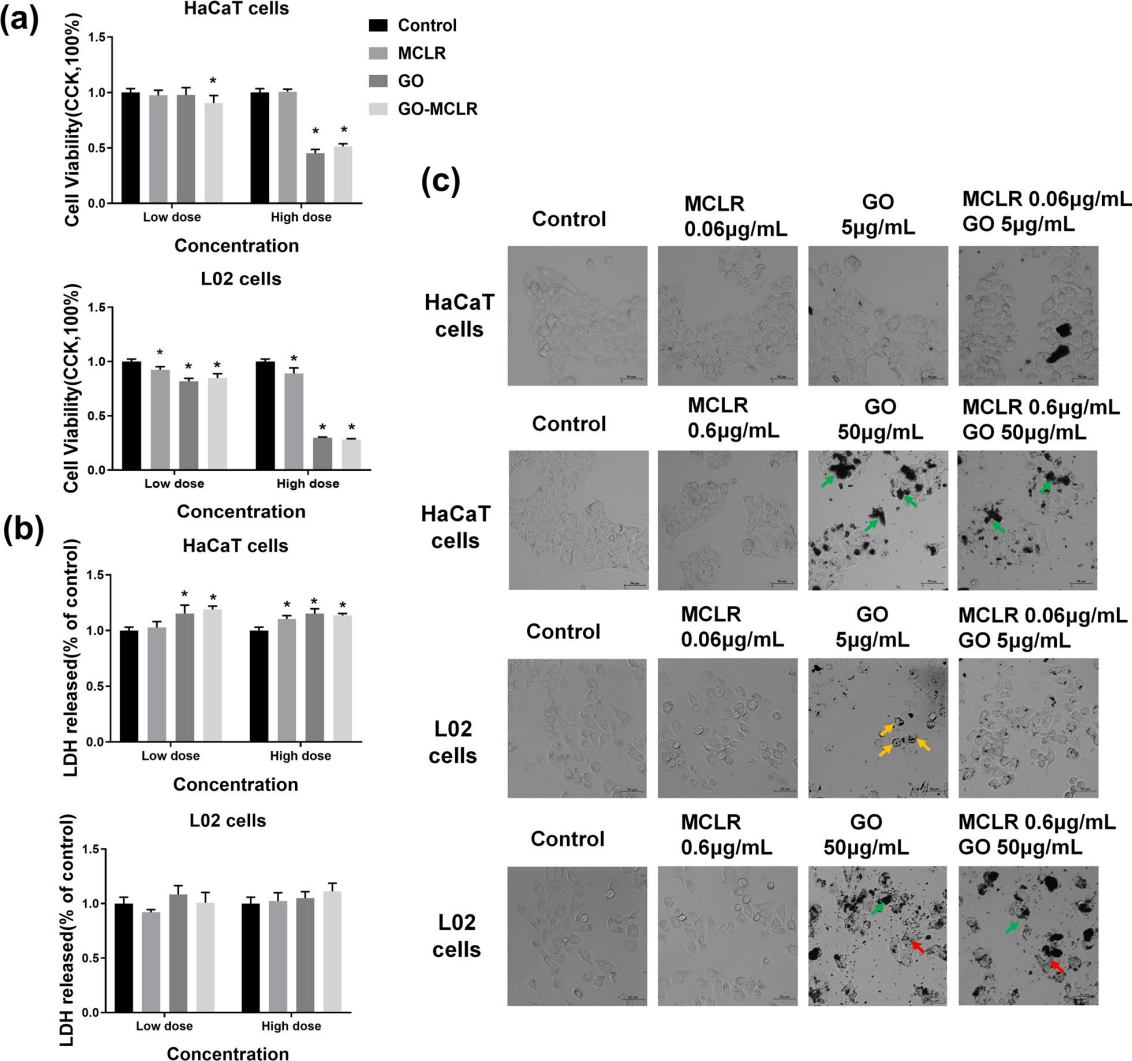
Cytotoxicity screening of GO, MCLR and GO-MCLR in HaCaT and L02 cells. HaCaT cells and L02 cells, seeded at the density of 1 × 10^5^ /mL, were respectively treated with GO (5 µg/mL and 50 µg/mL), MCLR (0.06 µg/mL and 0.6 µg/mL) and GO-MCLR (5 µg/mL + 0.06 µg/mL, 50 µg/mL + 0.6 µg/mL) for 24 h. **a** Use of a CCK-8 assay to assess the cells viability after exposure. The CCK-8 values in the control group was regarded as the 100% cell viability reference point. **b** LDH release amount representing the cell membrane integrity after exposure; **c** Cellular morphology of HaCaT and L02 cells under light microscope following exposure. *P < 0.05 versus Control. Green arrow: GO adhered to the cell surface; yellow arrow: the uptake of GO and GO-MCLR by cells; red arrow: cells rupture

Cytotoxicity studies were undertaken to obtain a provisional toxicological profiling of the materials in HaCaT cells and L02 cell lines, which represent target organs at risk under actual exposure of GO, MCLR and GO-MCLR. The cell viability of HaCaT cells and L02 cells treated with GO, MCLR and GO-MCLR complex for 24 h was assessed by CCK-8. As shown in Fig. 2a, single MCLR exposure has no significant effect on cell viability of HaCaT cells even when the exposure dose is as high as 0.6 µg/mL, whereas cell viability of HaCaT cells decreased to 45.3% compared with the control group after 50 µg/mL single GO treatment (*P* < 0.05). Notably, GO-MCLR significantly reduced HaCaT cells viability at the two dose levels adopted in this study. Compared with HaCaT cells, L02 cells were more sensitive to GO, MCLR and GO-MCLR, which exhibited by substantial reduction of the cell viability at both low and high doses. At high dose levels, the cell viability of L02 cells after GO and GO-MCLR exposure even decreased to 29.7% and 28.1%, respectively. To explore the effects of GO, MCLR and GO-MCLR on the membrane integrity of HaCaT cells and L02 cells, Lactate dehydrogenase (LDH) releases was detected. Compared with the control group, GO and GO-MCLR no matter at low dose or high dose induced more significant LDH release in HaCaT cells (*P* < 0.05), suggesting that GO-MCLR could lead to cell membrane damage similar to GO. On the contrary, the three materials did not affect the membrane integrity of L02 cells (Fig. 2b).

In order to directly reflect the cytotoxic effect of the materials, we observed and imaged the morphological changes of HaCaT and L02 cells after treatment with GO, MCLR and GO-MCLR, which exhibited in Fig. 2c. After MCLR treatment, no marked variation of cell morphology happened in HaCaT cell compared with the control group, but slight morphological changes like becoming round were observed in L02 cells. The number of HaCaT cells decreased after treating with high dose of GO (50 µg/mL), and a large amount of GO adhered to the cell surface indicated by the green arrow. GO-MCLR showed a more obvious effect on HaCaT cell morphology than GO, which was reflected in the shrinking and rounding of some cells at low dose, as well as the reduction of intercellular connections at high dose besides material attachment. From ACTEM images of L02 cells, it can be observed uptake of GO and GO-MCLR by L02 cells (yellow arrow) at low dose levels. With the rise of the exposure dose, the L02 cell morphology changed significantly, including the lower of cell number, loss of intercellular connections and cell rupture (red arrow).

### Induction of apoptosis by GO, MCLR and GO-MCLR in HaCaT cells and L02 cells

**Fig. 3:**
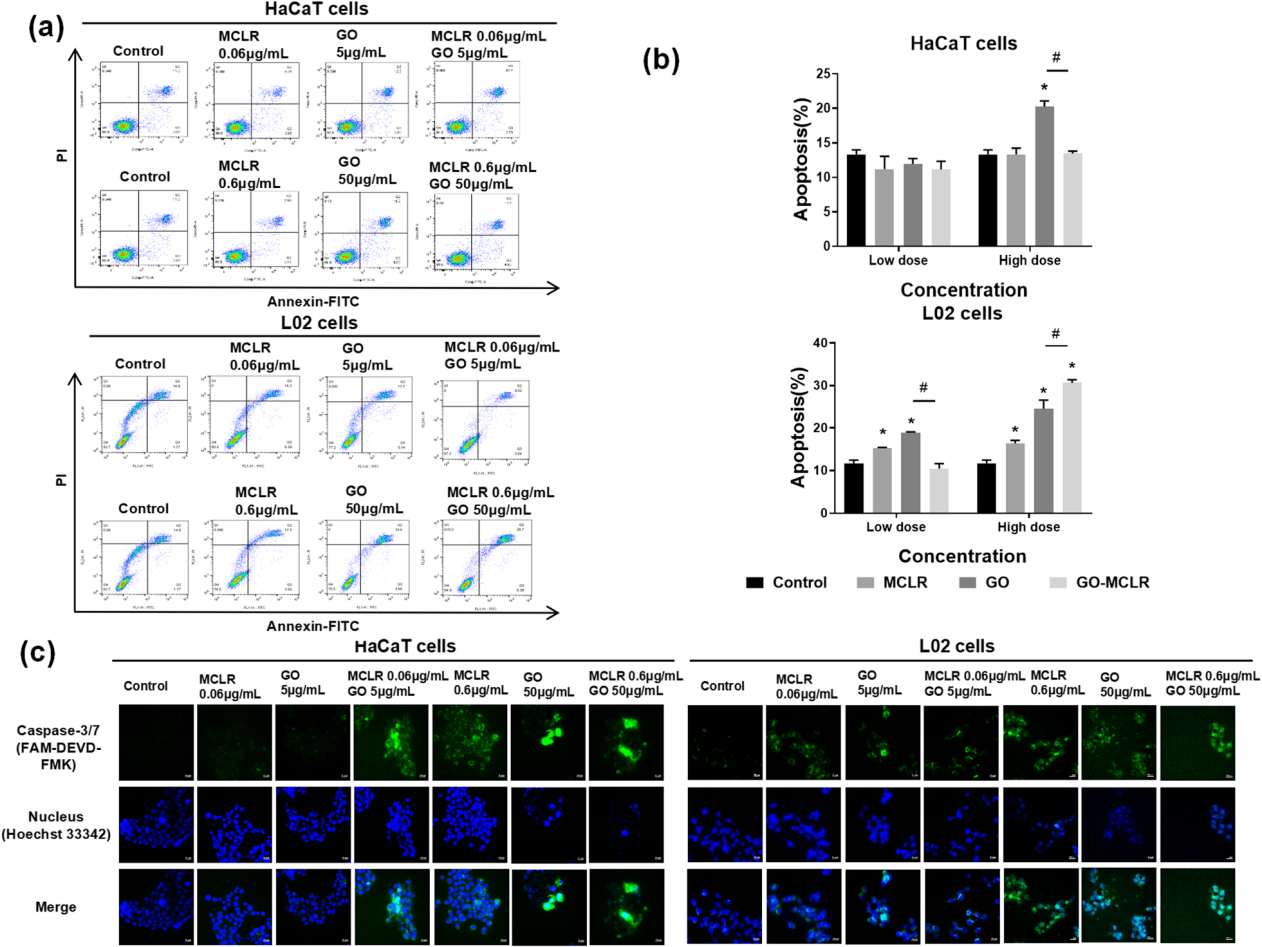
Apoptosis caused by GO, MCLR and GO-MCLR in HaCaT and L02 cells. HaCaT and L02 cells were cultured in 12-well culture dishes to confluence and exposed to GO (5 µg/mL and 50 µg/mL), MCLR (0.06 µg/mL and 0.6 µg/mL) and GO-MCLR (5 µg/mL + 0.06 µg/mL, 50 µg/mL + 0.6 µg/mL) up to 24 h. Then harvested cells were gently mixed with 300 μL Annexin V-FITC binding solution and 5 µL Annexin V-FITC, and incubated for 15 min at room temperature, away from light. 5 min before detection, 5 µL PI and 200 μL Annexin V-FITC binding solution were added. **a** After materials treatments and Annexin V-FITC/PI staining, cells apoptotic proportion were determined by flow cytometry. FITC (FL1) and PI (FL3) channels were selected, and 10,000 cells were analyzed per sample. **b** Quantitative analysis of proportion by flow cytometry, *P < 0.05 versus Control, #*P* < 0.05 versus GO group. **c** Confocal microscopy images showing GO, MCLR and GO-MCLR-induced caspases 3/7 expression in HaCaT and L02 cells cells. HaCaT/L02 cells were seeded on 24-well slides with a cell density of 4 × 10^4^ cells/mL,and then were treated with GO, MCLR and GO-MCLR of different concentrations for 24 h and washed with PBS. Cells were incubated with the prepared FLICA caspase 3/7 substrates for 1 h in the dark and rinsed 3 times with the buffer solution. Cell nuclei were stained with Hoechst 33,342

To explore the effects of GO, MCLR and GO-MCLR on apoptosis rates of HaCaT cells and L02 cells respectively, the apoptosis of cells in each group were detected by flow cytometry. According to flow cytometry results which shown in Fig. 3a, b, the three materials had no notable impact on the apoptosis of HaCaT cells at low dose, and at high dose, only GO induced significant apoptosis at high dose with the apoptosis rate increasing to 20.3%. The L02 cells showed a stronger apoptosis level after 24 h exposure of GO, MCLR and GO-MCLR than HaCaT cells. Low dose MCLR could bring about increase of L02 cell apoptosis rate (*P* < 0.05), and when the concentration of MCLR was 0.6 µg/mL, L02 cell apoptosis rate added up to 16.4%. Similarly, treatment of 50 µg/mL GO caused that the apoptosis rate of L02 cells was further increased to 24.5%, on the basis that 5 µg/mL GO had significantly induced L02 cells apoptosis. After GO adsorption of MCLR, apoptosis caused by GO could be markedly alleviated at low dose level. Interestingly, high-dose GO-MCLR promoted the apoptosis rate of L02 cells to increase from 24.5% induced by GO to 30.6%, which was worth further discussion.

Apoptosis usually depends on the activation of the Caspase family, of which Caspase-3 and Caspase-7 are important executors. Therefore, the activation of caspase-3/7 under different treatments were measured. For HaCaT cells, there was no obvious fluorescence after low-dose single MCLR and single GO treatment, while observable increase in fluorescence intensity was existed in HaCaT cells exposed to GO-MCLR at low dose (MCLR of 0.06 µg/mL and GO of 5 µg/mL). At high dose levels, all three materials at high dose induced the addition of fluorescence intensity in HaCaT cells suggesting the activation of Caspase-3/7, and the fluorescence intensity was more obvious in GO and GO-MCLR treatment groups than single MCLR treatment. The change trend of Caspase-3/7 in L02 cells after treatment was consistent with that of apoptosis rate. The clear fluorescence enhancements were observed in all treatment groups, and more significant activation of Caspase-3/7 appeared with the increase of dose (Fig. 3c).

### Induction of ferroptosis by GO, MCLR and GO-MCLR in HaCaT and L02 cells

**Fig. 4:**
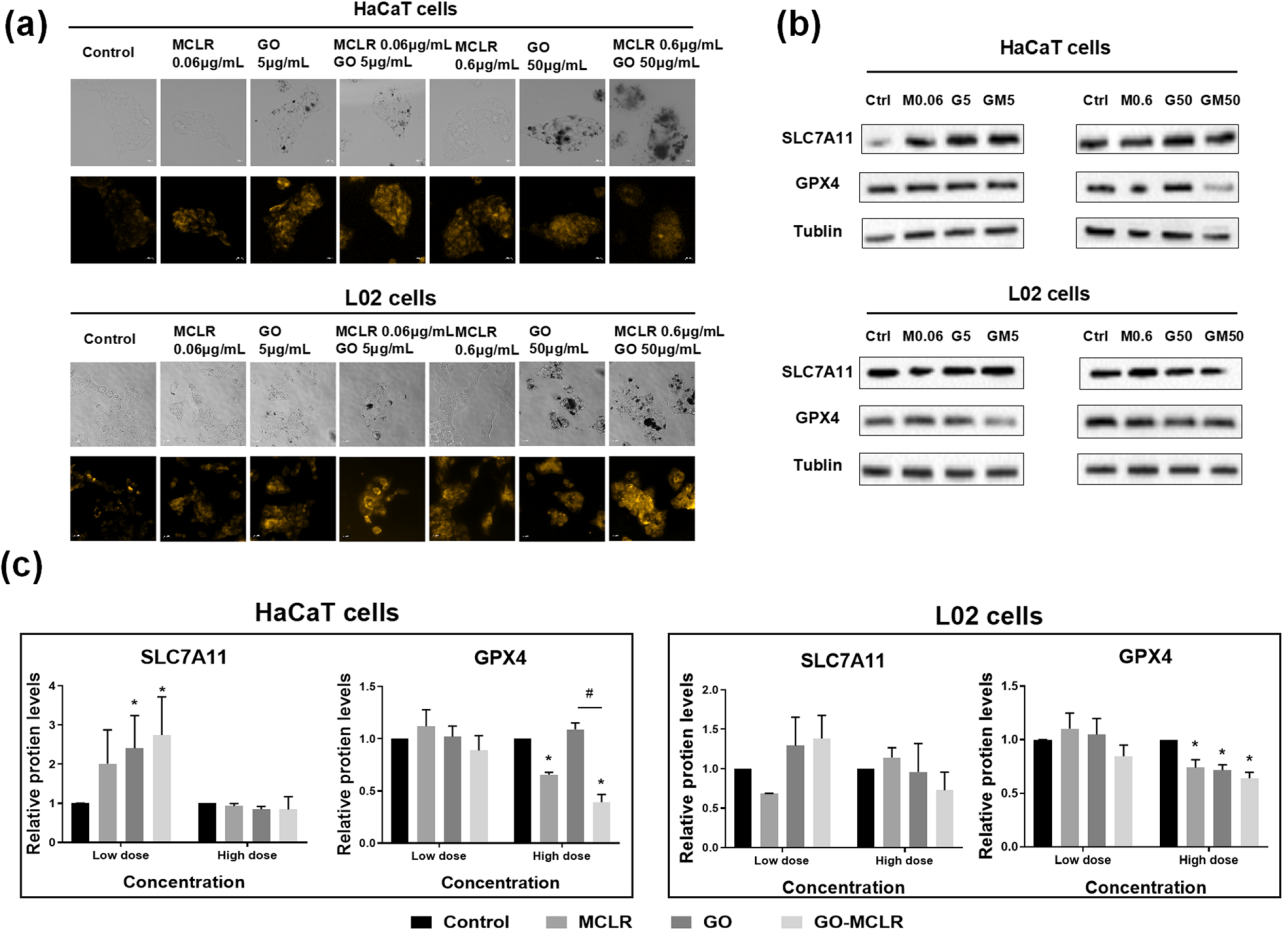
Ferroptosis caused by GO, MCLR and GO-MCLR in HaCaT and L02 cells. HaCaT and L02 cells were co-incubated with GO (5 µg/mL and 50 µg/mL), MCLR (0.06 µg/mL and 0.6 µg/mL) and GO-MCLR complex (5 µg/mL + 0.06 µg/mL, 50 µg/mL + 0.6 µg/mL) suspensions for 24 h. **a** After exposure, HaCaT/L02 cells were incubated with the FerroOrange ferrous ion fluorescence probe for 1 h keeping out light. The content of Fe^2+^ in the cells were imaged using the g-excitation filter in fluorescence microscopy. The stronger the fluorescence, the more Fe^2+^ accumulated in the cells. **b** The level of SLC7A11 and GPX4 expression were analyzed by Western blot. M0.06: MCLR 0.06 µg/mL; G5: GO 5 µg/mL; GM5: GO-MCLR 5 µg/mL + 0.06 µg/mL; M0.6: MCLR 0.6 µg/mL; G50: GO 50 µg/mL; GM50: GO-MCLR 50 µg/mL + 0.6 µg/mL. **c** Band intensities were quantified and normalized to Tublin. *P < 0.05 versus Control, #*P* < 0.05 versus GO group

In addition to apoptosis, ferroptosis is also one of the programmed death mechanisms that has attracted much attention in recent years. The Fe^2+^ situation in HaCaT and L02 cells after different exposure were shown in Fig. 4a. The fluorescence intensity in HaCaT cells became stronger in a dose-dependent manner after MCLR exposure compared to weak fluorescence in the control group. It should be noted that fluorescence intensity of HaCaT cells increased more significantly after low-dose GO and GO-MCLR treatment than after high-dose treatment, which may be due to the occurrence of Fenton reaction. L02 cells in the control group showed no obvious fluorescence, indicating the content of Fe^2+^ in cells under normal conditions. Fluorescence images showed that all three toxic materials could cause Fe^2+^ accumulation in L02 cells dose-dependently. By comparing the Fe^2+^ fluorescence intensity of L02 cells treated with the three materials, it was found that GO-MCLR could induce more serious Fe^2+^ accumulation in L02 cells than GO or MCLR. To further explore the occurrence of ferroptosis in cells, the expressions of SLC7A11 and GPX4 proteins related to ferroptosis were detected by Western Blot (Fig. 4b, c). At low doses of exposure, only the upstream protein SLC7A11 expressions of HaCaT cells treated with GO and GO-MCLR were dramatically up-regulated. However, high dose exposure of poisonous materials effectively reduced the expression of GPX4 (a key downstream protein of ferroptosis) in HaCaT cells and L02 cells, although it had no effect on the SLC7A11. In summary, we found that exposure to high concentrations of MCLR, GO and GO-MCLR leaded to ferroptosis in both two cells, and GO-MCLR treatment remarkably activated the ferroptosis-related SLC7A11/ASCL4/GPX4 pathway in HaCaT cells and L02 cells.

### Intracellular oxidative stress caused by GO, MCLR and GO-MCLR in HaCaT cells and L02 cells

**Fig. 5:**
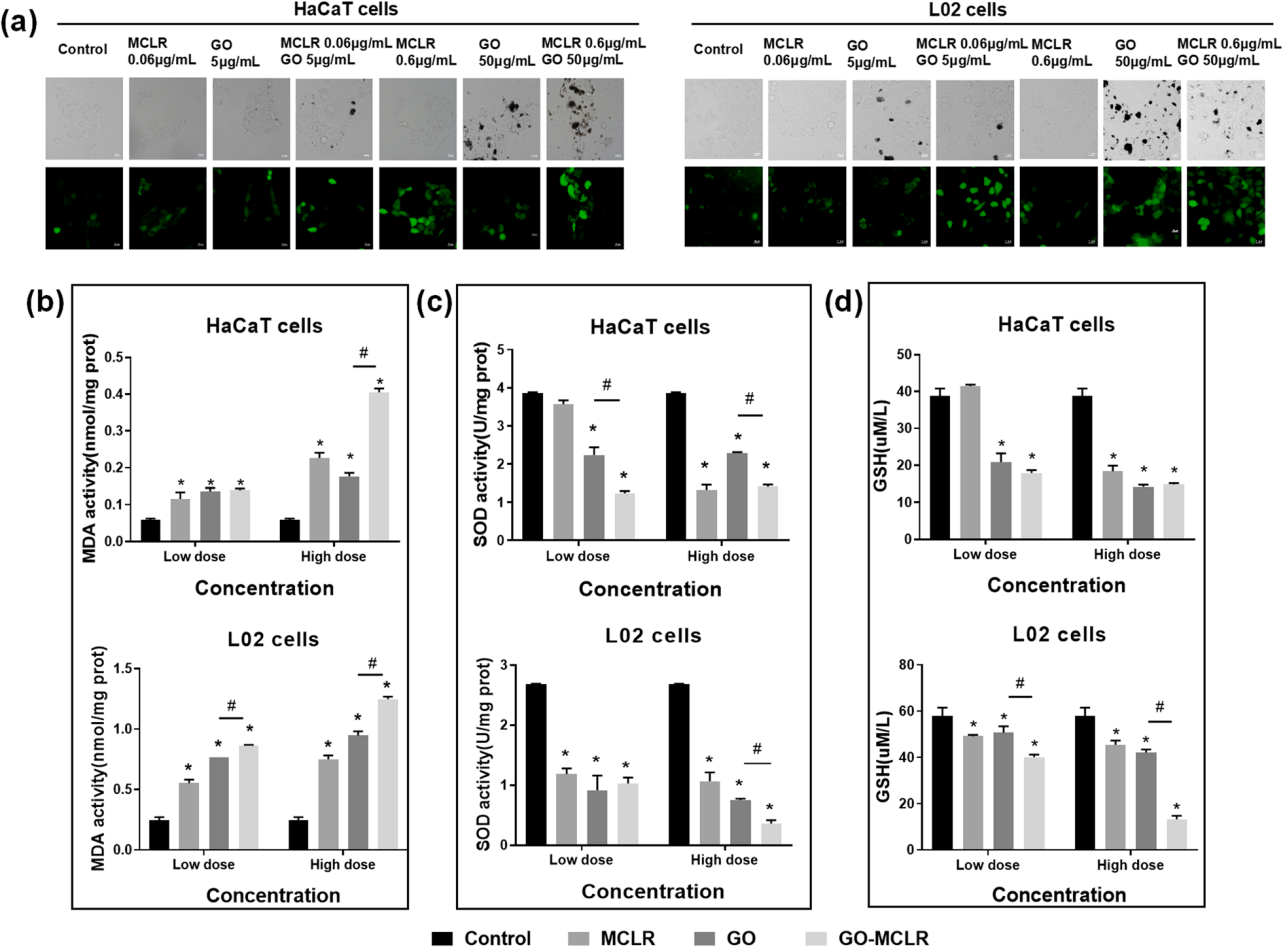
The role of ROS production and oxidative stress in Programmed cell death induced by GO, MCLR and GO-MCLR. HaCaT cells and L02 cells at logarithmic growth stage were inoculated in 12-well plates at a density of 1 × 10^5^/mL. **a** Intracellular ROS production identified by DCFH-DA in HaCaT and L02 cells treated with GO (5 µg/mL and 50 µg/mL), MCLR (0.06 µg/mL and 0.6 µg/mL) and GO-MCLR complex (5 µg/mL + 0.06 µg/mL, 50 µg/mL + 0.6 µg/mL) for 24 h using fluorescence microscopy. Excitation wavelength of 488 nm and emission wavelength of 525 nm for the incidence of intracellular ROS. The activities of SOD (**b**), MDA (**c**) and GSH (**d**) in treated cells were detected by corresponding kits. *P < 0.05 versus Control, #*P* < 0.05 versus GO group

For nanomaterials including GO, the excessive generation of ROS and the following intracellular oxidative stress are the central link in inducing cell damage, and also one of the mechanisms of MCLR cytotoxicity. We first observed the changes of ROS in treated HaCaT cells and L02 cells by DCFH-DA staining, as shown in Fig. 5a. GO, MCLR and GO-MCLR all caused enhancement of fluorescence intensity in these two cells, showing the accumulation of intracellular ROS, and the higher the dose of exposure, the more ROS production. Compared with GO infection, GO-MCLR stronger fluorescence intensity in both HaCaT cells and L02 cells were detected, which suggested that GO-MCLR could induce more serious ROS overproduction than GO. In addition, intracellular oxidative stress caused by excessive accumulation of ROS were determined by the contents of Superoxide dismutase (SOD), Malondialdehyde (MDA) and glutathione (GSH) in HaCaT cells and L02 cells (Fig. 5b). By analyzing the contents of SOD, MDA and GSH in HaCaT cells of different treatment groups, it was found that GO, MCLR and GO-MCLR all lead to a significant decrease of SOD, a notable increase of MDA, and a remarkable exhausting of GSH in a dose-dependent manner. Similar but relatively stronger results were observed in L02 cells, which meant that oxidative stress induced by these three materials was more intense in L02 cells than in HaCaT cells. Notably, both HaCaT cells and L02 cells treated with GO-MCLR have lowest SOD content, as well as highest MDA content among cells treated with GO and MCLR, indicating that the GO-MCLR composite induced more significant intracellular oxidative stress than GO or MCLR alone.

### Mitochondrial injury induced by GO, MCLR and GO-MCLR in HaCaT cells and L02 cells

**Fig. 6:**
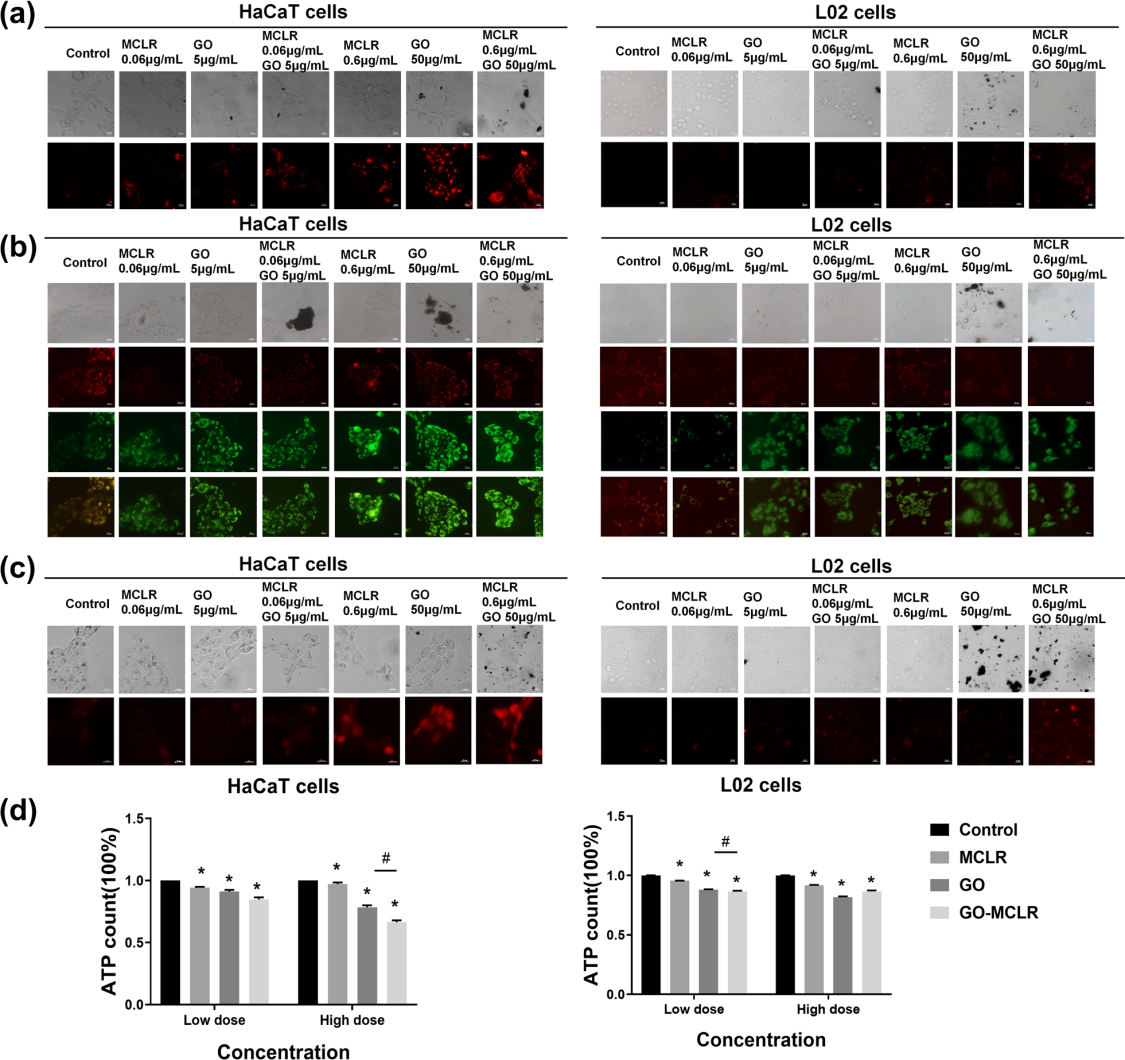
Assessment of mitochondrial injury induced by GO, MCLR and GO-MCLR in HaCaT and L02 cells. **a** Mitochondrial ROS were detected by MitoSOX. The HaCaT cells and L02 cells exposed to diverse concentrations of GO, MCLR and GO-MCLR for 24 h were co-incubated with MitoSOX probe for 10 min at a concentration of 5 µM and a dilution ratio of 1:1000. Then, the cells were rinsed 3 times with PBS preheated at 37 ℃. MtROS production levels were observed with Zeiss microscope at excitation wavelength of 510 nm and emission wavelength of 580 nm, and red fluorescence indicated the generation of mtROS. **b** MMP identified by JC-1 probe. The fluorescent images were obtained by fluorescence microscope at excitation wavelength of 514 nm and emission wavelength of 529 nm for JC-1 monomer, excitation wavelength of 585 nm and emission wavelength of 590 nm for JC-1 aggregates. **c** Mitochondrial ATP generations in HaCaT and L02 cells treated by GO (5 µg/mL and 50 µg/mL), MCLR (0.06 µg/mL and 0.6 µg/mL) and GO-MCLR complex (5 µg/mL + 0.06 µg/mL, 50 µg/mL + 0.6 µg/mL) were measured by the ATP assay kit using a microplate luminescence detector. **d** The level of Ca^2+^ were identified in HaCaT/L02 cells stained with Fluo-3AM fluorescence probe. The infected cells were added with 1 mL Fluo-3AM probe diluted in serum-free medium (1:1000) and incubated for 1 h. Intracellular Ca^2+^ concentration was observed at excitation wavelength 506 nm and emission wavelength 526 nm by fluorescence microscopy. *P < 0.05 versus Control, #*P* < 0.05 versus GO group

As the main site for the production of reactive oxygen free radicals, mitochondria will have dysfunction when they are under stress, hypoxia and other undesirable states. Firstly, MitoSOX probe was used to detect the ROS production in mitochondria so as to assess the effects of different doses of MCLR, GO and GO-MCLR on mitochondrial homeostasis of HaCaT cells and L02 cells. As shown in Fig. 6a, there were no obvious fluorescence in HaCaT cells at low dose material exposure, but the fluorescence intensity of HaCaT cells statistically significant increased after high dose of material exposure, among which the enhancement of mitochondrial ROS attributed to GO-MCLR exposure was the most forceful. While low and high doses of MCLR, GO and GO-MCLR had no evident effect on the mitochondrial ROS of L02 cells. Then membrane potential (MMP), one of the indicators of mitochondrial function, was detected in treated HaCaT cells and L02 cells. Through fluorescence staining (Fig. 6b), strong red fluorescence in HaCaT cells without any treatment was observed, indicating that JC-1 exists in HaCaT cells as a polymer under normal conditions. Compared with the control group, HaCaT cells in different treatment groups showed weak red fluorescence but strong green fluorescence. For the same material, the higher the dose of exposure, the more definite the green fluorescence, which also meant a more serious decrease in MMP. For L02 cells, varying degrees of mitochondrial MMP changes caused by GO, MCLR and GO-MCLR were found, but there was no dose-dependence. Importantly, the change of green fluorescence in HaCaT cells was higher than that in L02 cells at the same dose of the same material, suggesting that MMP decreased significantly in HaCaT cells than that in L02 cells.

After the dysfunction of mitochondria, its functional product adenosine triphosphate (ATP) will also change. Therefore, we then measured ATP content in HaCaT and L02 cells respectively after exposure. It was found that after 24 h of exposure, ATP levels in all treatment groups, both HaCaT and L02 cells, were significantly reduced when comparing with the control group (*P* < 0.05) (Fig. 6d). Among them, ATP content of HaCaT cells decreased by 21.6% and 33.5% under high-dose GO exposure and high-dose GO-MCLR exposure respectively. There was also a statistical difference between the two, which meant that the ATP synthesis disorder in HaCaT cells induced by the GO-MCLR complex more serious than that induced by GO. Similarly, in the low-concentration GO-MCLR group, ATP level in L02 cells was also notably down-regulated compared with GO exposure (*P* < 0.05). Mitochondrial calcium overload caused by increased intracellular Ca^2+^ levels result in changes in mitochondrial membrane permeability. Finally, Fluo-3 AM probes were used to reflect intracellular Ca^2+^ levels which was proportional to the fluorescence intensity in Fig. 6c. High-dose GO, MCLR and GO-MCLR caused intracellular Ca^2+^ overload in HaCaT cells compared with the control group, while there was no significant difference in fluorescence intensity of L02 cells in varying groups, and only slight intracellular Ca^2+^ upregulating was accompanied by high-dose GO-MCLR exposure. In summary, it was found that high dose of GO, MCLR and GO-MCLR induced strong mitochondrial injury in HaCaT cells, while the degree of mitochondrial damage in L02 cells was relatively low under the same treatment. In addition, multiple evidences suggested that more severe mitochondrial injury occurred after GO-MCLR exposure compared to single material exposure.

### Changes of specific injury induced by MCLR before and after GO adsorption

**Fig. 7:**
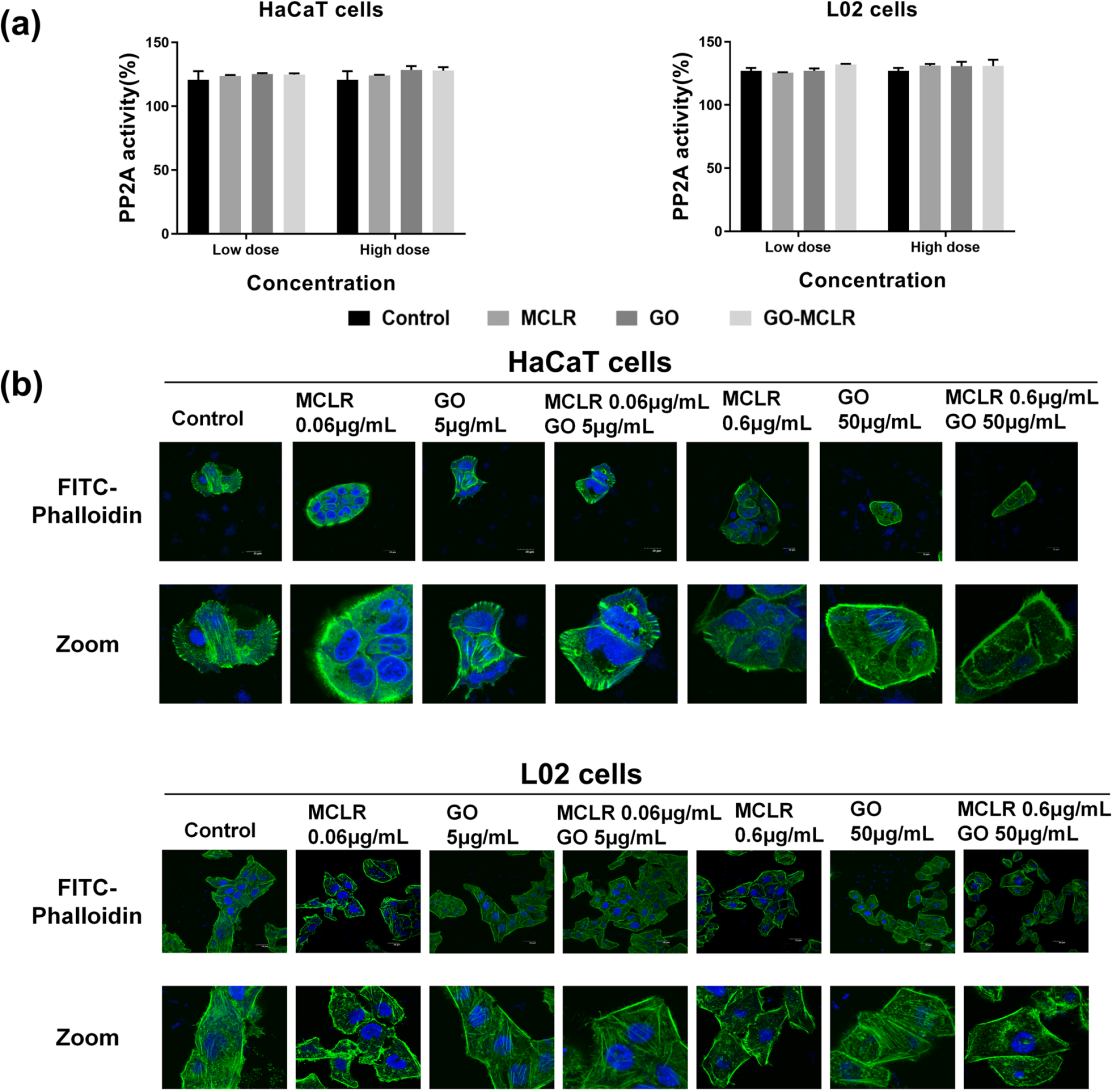
Identification of specific injury induced by MCLR before and after GO adsorption. HaCaT and L02 cells were co-incubated with GO (5 µg/mL and 50 µg/mL), MCLR (0.06 µg/mL and 0.6 µg/mL) and GO-MCLR complex (5 µg/mL + 0.06 µg/mL, 50 µg/mL + 0.6 µg/mL) suspensions for 24 h. **a** After 24 h of treatment, the cells culture medium was collected and analyzed for PP2A levels. **b** Cells cytoskeleton damage was determined via FITC-Phallodin staining. After 24 h of exposure, the cells were fixed with 4% paraformaldehyde dissolved in PBS for 10 min and treated at room temperature for 5 min with 0.5%Triton X-100 dissolved in PBS. 200 µL FITC-Phallodin staining solution was added and incubated for 30 min at room temperature without light. 100 µL anti-fluorescence quenching agent containing DAPI was added, and the staining results were imaged under laser confocal microscopy. The FITC (Ex/Em = 492/518 nm) and DAPI (Ex/Em = 364/454 nm) excitation/emission filters were selected

Many studies believe that cytotoxicity of MCLR can be attributed to PP2A activity inhibition and F-Actin-mediated cytoskeletal damage, so the above two specific damage indicators were determined. By analyzing the activity of PP2A in HaCaT and L02 cells, it was found that there was no significant change in PP2A activity of cells treated with the three materials for 24 h compared with the control group (*P* > 0.05), and the performance of the two cells was consistent (Fig. 7a). Considering cell viability, the concentration of MCLR selected for this study was 0.6 µg/mL (0.6 µM), which was too low to rouse visible variation of PP2A activity. Confocal laser was used to further observe the effects of the three materials on F-Actin. As we can see from Fig. 7b, different from the clear and orderly microfilaments of HaCaT cells in the control group, microfilaments were broken with 0.06 µg/mL MCLR treatment, and fluorescence brightness was further reduced at high dose, suggesting that microfilaments were depolymerized and dispersed. When HaCaT cells were exposed to GO-MCLR, a small number of microfilaments were observed to be retained at low dose, although microfilaments depolymerization occurred at high dose of GO-MCLR. For L02 cells, F-actin brightness decreases, microfilament depolymerization and the appearance of filopodia at the polar end of the cell were found in L02 cells induced by MCLR at both doses. Similar to the results in HaCaT cells, some microfilament structures were still retained in L02 cells after low-dose GO-MCLR treatment. While the microfilament damage was not improved after high-dose GO-MCLR exposure, compared with the same dose MCLR exposure. In summary, MCLR could induce microfilament damage in both HaCaT cells and L02 cells, and GO-MCLR alleviated cytoskeletal damage in comparison with MCLR at low dose, which may be related to reducing of free MCLR in cells thanks for GO adsorption.

## Discussion

As an attractive and promising nanomaterial, it is inevitable for GO to enter the aquatic environment through different routes including environmental restoration, ecological cycle and so on [27]. In addition, MCLR which has been identified by several studies as a powerful tumor promoter, is a cyanobacterial toxin with the widest distribution, maximum production and most serious harm in the aquatic environment [21]. When GO absorbed MCLR, the possible toxic transformation of GO is still unclear. Therefore, in this study, we systematically evaluated the adsorption behavior of GO for MCLR in the aquatic environment, the subsequent changes in material biosafety and its potential mechanism. The GO concentrations in the pre-experiment that resulted in half cell death (50 µg/mL) and no significant cytotoxic effect (5 µg/mL) were respectively set as the high and low doses in this study, then based on adsorption kinetics model of GO for MCLR (Additional file 1: Figure S2), the adsorption amount of GO to MCLR when adsorption equilibrium was about 12 µg/mg, so 0.6 µg/mL and 0.06 µg/mL MCLR was selected to subsequently prepare GO-MCLR complexes. According to the adsorption isotherm of GO to MCLR (Additional file 1: Figure S3), although there were still a small number of free MCLR, most particles in the solution (50 µg/mL GO + 0.6 µg/mL MCLR) under such conditions already exist in the form of GO-MCLR complex.

Huge specific surface area, developed microporous structure and abundant surface functional groups determine the excellent adsorption performance of GO [13]. Consistent with the expectation, the quantitative adsorption of GO for MCLR showed that the adsorption amount enhanced rapidly in a short time when the pH was neutral. According to Langmuir adsorption theory, when adsorbents occupy the adsorption site, no further adsorption will take place at that site [28]. This can well explain the process shown in Fig. 1a that, as time goes on, the adsorption rate of GO for MCLR decreases gradually until adsorption equilibrium after rapid adsorption in the early stage. The ACTEM images visually showed the adsorption of GO on MCLR, which was manifested as fusiform MCLR attached to GO surface folds, but the adsorption mechanism needed exploring. The π–π conjugation has been used to explain the adsorption of organic matter with C=C double bond or benzene ring on graphene surface [29]. As MCLR is a class of bioactive cyclic heptapeptides with conjugated double bonds, benzene rings and methoxy groups, there are sufficient reasons for us to speculate that the main molecular force between MCLR and GO is π–π conjugation. In addition, oxygen-containing groups on the surface of GO, for example, hydroxyl, can form hydrogen bonds with the hydroxyl group of MCLR. Huang et al*.* found that the original carbon material containing more hydroxyl groups adsorbed more MCLR due to the formation of hydrogen bonds between the positively charged arginine side chain of MCLR and the negatively charged carbon surface [30]. To further elucidate the adsorption mechanism, Raman, FITR and XPS spectra of GO and GO-MCLR were compared. In Raman spectrum the value of ID/IG reflects the defect degree of GO. So, the increase of ID/IG in this study indicated lower graphitization and higher disorder degree of GO after MCLR adsorption. Both FTIR and XPS spectrum showed that most oxygen-containing groups have been removed after GO adsorbed MCLR. The above results suggested that the main mechanism of GO adsorption of MCLR was hydrogen bonding and π–π conjugation. After the adsorption of MCLR, the oxygen-containing functional groups on GO were replaced, the defect sites were occupied, and the disorder degree of GO increased.

As previously mentioned, GO could efficiently and stably adsorb MCLR and form a new complex GO-MCLR. In order to find out whether there is a difference in toxicity between GO-MCLR and the single material, this study took HaCaT cells and L02 cells as in vitro cell models referring to actual contact and material target organs, then assessed the toxicity behavior and characteristics of materials. CCK-8 showed that GO and GO-MCLR induced similar reduction of cell viability in both HaCaT cells and L02 cells. Importantly, MCLR, GO and GO-MCLR induced more severe activity inhibition of L02 cells at the same dose, which may be related to with strong adaptability and survival ability of skin keratinocytes. Indeed, this result is consistent with previous studies that the primary target organ of MCLR and GO after exposure is the liver, while cutaneous toxicity is rarely reported [31, 32]. LDH is one of the intracytoplasmic enzymes in living cells and is often used to evaluate the integrity of cell membranes. Li et al*.* proved that unmodified GO produced significant toxicity to macrophages, which were notably eased by coating GO surface with bovine serum albumin to form a protein crown [33]. But here, GO greatly increased LDH release in HaCaT cells at both dose levels, and the GO-MCLR complex did not significantly alleviate cell membrane integrity damage caused by GO. As mentioned earlier, morphological observation of the adsorbed material found that MCLR attached to part of the fold and edge on GO, not fully covering the sharp edge. The GO-MCLR failed to form a complete protein crown, and there were no aggravating or ease of cell membrane injury on cells treated with GO-MCLR. Interestingly, none of the three materials affected LDH release from L02 cells, which was largely attributed to programmed cell death. Even the membrane blebbing occurs during apoptosis, but the structure of cell membrane is still intact without content overflow [34]. Similarly, the morphology of ferroptosis cells does not involve cell membrane rupture [35]. While pyroptosis is usually accompanied by GSDMD—induced pore formation and eventual membrane lysis [36]. In this study, through exploring the forms of programmed cell death, it was found that apoptosis and ferroptosis were the main forms of cell death caused by materials, and pyroptosis was observed but not significant, which reasonably explained the insignificant LDH release of L02 cells under the effects of the three materials. Further cell morphology observation demonstrated that high-dose GO and GO-MCLR caused a decrease in cell number and intercellular connections, and a large amount of GO was attached to the cell surface. Consistent with CCK-8 results, the morphological changes of L02 cells were more significant than those of HaCaT cells. In summary, GO-MCLR could cause cytotoxic effects similar to GO. From the perspective of cell viability and morphology, L02 cells were more sensitive to materials than HaCaT cells, but the cell membrane integrity of HaCaT cells was significantly damaged.

The differences in injury patterns between HaCaT and L02 cells suggested other potential toxic effects of MCLR, GO and GO-MCLR. According to the morphological changes, it could be inferred that materials might exert cytotoxicity through a series of gene-controlled programmed death, instead of cell necrosis. The spontaneous and orderly death controlled by gene that may occur in cells stimulated by exogenous factors to keep the internal environment stable is called apoptosis. It is known that apoptosis is a programmed death that may be involved both in toxicity studies of GO and MCLR [37, 38]. Therefore, apoptosis was first selected to be discussed in this study. In terms of HaCaT cells, high doses of MCLR, GO and GO-MCLR, significantly activated apoptosis-related protein caspase-3/7 in HaCaT cells [39], but flow cytometry results did not show strong apoptosis except for high dose of GO. The possible reason was that due to its indigestion characteristics, the long digestion time of HaCaT cells during the flow cytometry assay of apoptosis affected the effective binding of fluorescent probes. For L02 cells, both the result of Caspase-3/7 and flow cytometry indicated the occurrence of apoptosis after MCLR, GO and GO-MCLR treatment. Interestingly, GO-MCLR reversed cell apoptosis induced by MCLR and GO at low dose, but apoptosis was further promoted in the high-dose GO-MCLR group. There are two reasons for this phenomenon, one is that at low doses, the addition of MCLR can partially alleviate the uptake of GO by L02 cells which leads to apoptosis. This is similar to previous results. The study by Zhang showed that the adsorption of BSA on CNTs significantly reduced its cellular uptake, thus effectively improving the biosafety of CNTs [40]. The other reason is that GO can alleviate the damage of MCLR at low dose to L02 cells cytoskeleton which has been proved to aggravate the apoptosis caused by exogenous substances.

Cell swelling and cell membrane rupture are the main morphological characteristics of pyroptosis [41]. So, the increase of LDH observed in HaCaT cells drove us to detect cell pyroptosis. However, related indicators including GSDMD and pyroptosis rate all proved that pyroptosis was not the main form of cell damage induced by MCLR, GO and GO-MCLR, which prompted us to explore another potential programmed cell death (Additional file 1: Figure S1). Recently, ferroptosis has attracted much attention as a new programmed death and several types of nanomaterials are known to cause It [42]. Wu et al. found that nitrogen-doped graphene quantum dots could induce iron overload and ferroptosis in BV2 cells [43]. Although no studies on ferroptosis of MCLR have been reported, many toxic mechanisms of MCLR, such as excessive ROS generation and lipid peroxidation, are related to ferroptosis. In this study, exposure to MCLR, GO and GO-MCLR resulted in accumulation of Fe^2+^ in both HaCaT cells and L02 cells, indicating that ferroptosis also partially contributed to the cytotoxicity induced by MCLR, GO and GO-MCLR. In L02 cells, GO-MCLR caused stronger Fe^2+^ imbalance than the same dose of GO and MCLR. While in HaCaT cells, the fluorescence intensity of Fe^2+^ decreased in high-dose GO-MCLR group compared with single material group, which might be linked to the Fenton reaction causing oxidation of Fe^2+^ into Fe^3+^ [44]. In addition, current studies suggest that the inactivation of GPX4 and SLC7A11 can promote ferroptosis. Zhang et al. showed that carbon tubes, GO and other iron-free nanomaterials could still induce the increase of Fe^2+^ and the down-regulation of GPX4 gene in HUVECs cells, thus inducing ferroptosis [45]. Our study found that the expressions of GPX4 in HaCaT and L02 cells were significantly down-regulated when exposed to high-dose materials, further indicating the occurrence of intracellular ferroptosis. The feedback regulation induced by cellular stress reasonably explained the up-regulation of SLC7A11 in HaCaT cells after low-dose GO and GO-MCLR treatment. Similar to our results, Lei et al*.* found that ionizing radiation (IR) induced ferroptosis in cancer cells. At the same time, significantly upregulation of SLC7A11 was observed, the authors speculated that IR-induced expression of SLC7A11 may function as a negative feedback loop to restore cell survival upon IR [46].

On the basis of programmed death, the mechanism of toxicity induced by materials was further discussed. ROS production is the result of a combination of cellular aerobic respiration and oxidative phosphorylation, reflecting the imbalance between the production of active substances and antioxidant defense. Under normal physiological conditions, ROS can be removed by endogenous antioxidant system in time to maintain intracellular homeostasis. Once this balance is broken, ROS would accumulate excessively and oxidative stress happen in the cell [47]. It has been reported that both GO and MCLR can induce ROS production in different types of cells, and high levels of ROS in cells directly or indirectly participate in programmed cell death [48, 49]. Here, we found that GO, MCLR and GO-MCLR all caused dose-dependent increase of ROS, as well as changes of SOD, MDA and GSH content in HaCaT cells and L02 cells. All the above indicators indicated the occurrence of oxidative stress after materials treatment. Meanwhile, GO-MCLR has been shown to cause more intense oxidative stress than single GO and MCLR exposure. Compared with MCLR, GO-MCLR enter cells more easily with the help of GO. Consistent with our results, Wu et al*.* found that the co-exposure of nano-TiO_2_ and MCLR aggravated the swimming and social behavior disorders of zebrafish by inducing severe oxidative stress, which was ascribed to higher biological uptake of MCLR promoted by nanomaterials [50]. In addition, the reduction of oxidation degree and higher disorder are main reasons for more obvious oxidative stress of GO-MCLR than GO. Zhao's study compared the oxidation degree and corresponding toxicity of GO before and after light transformation, and found that the oxidation degree of GO after UV-transformation was lower, and the following high conductivity further caused more intense membrane damage and oxidative stress [51]. The study of Zhang has proved that the reduction of disordered structure of GFNs was one of the possible reasons for the significant decrease in the potential toxicity of GFNs to *S. Obliquus* [52].

As the main site of aerobic respiration, mitochondria are important organelles for energy supply. Mitochondrial ROS (mtROS) can also play a role in signal transduction and thus participate in various biological processes [53]. It has been reported that mitochondrial oxidative imbalance and mitochondrial dysfunction are also important causes of programmed cell death. For example, the study of Liu also proved that mitochondrial vacuolation, decreased MMP and over-expressed mtROS caused by PM2.5 triggered cell apoptosis [54]. However, the role of mtROS and mitochondrial dysfunction in programmed cell death induced by GO and MCLR is still lacking. Our results showed that high-dose GO, MCLR and GO-MCLR greatly up-regulated the content of mtROS in HaCaT cells and induced mitochondrial oxidative stress. The mitochondrial functional indicators were further assessed after the accumulation of mitochondrial ROS was uncovered. We discovered that the MMP and ATP production capacity were significantly reduced after treatment with high dose materials, indicating the mitochondrial dysfunction of HaCaT cells. In addition, mitochondria can regulate energy metabolism and cell death by participating in intracellular calcium signal transduction and calcium homeostasis. In this study, the growing of Ca^2+^ level in HaCaT cells after GO, MCLR and GO-MCLR exposure suggest imbalance of calcium homeostasis. All these results indicated that severe mitochondrial injury had occurred in treated HaCaT cells, and mitochondrial dysfunction induced by GO-MCLR was more serious than that caused by single material exposure. Differently, only mitochondrial dysfunction was observed in L02 cells after exposure, without accumulation of mtROS and imbalance of calcium homeostasis.

In addition to the mechanism of cytotoxicity induced by both GO and MCLR, we further focused on the mechanism of MCLR specific toxicity. On the one hand, MCLR can directly bind to PP2A/C subunit to inhibit the activity of PP2A, which is one of the toxic mechanisms of MCLR [55]. Possibly due to low exposure concentrations, neither MCLR nor GO-MCLR showed inhibition of PP2A activity in either cell type of this study. Relevant research showed that at the dose level of 1 μg/mL, which was higher than the dose used in this study, MCLR still failed to induce significant changes in cell viability and apoptotic proteins of Sertoli cells [56]. On the other hand, cytoskeleton destruction and reorganization are another important mechanism of cells damage induced by MCLR, including tubulin and microfilament phosphorylation, F-actin decomposition and stress fiber shortening [57]. The results of Avci pointed that when the cytoskeleton was reorganized after exogenous stimulation, the migration behavior of cells changed and the exogenous induced apoptosis was intensified [58]. In our study, MCLR resulted in the diffusion of F-Actin in HaCaT cells and L02 at a dose level of 0.06 µg/mL, showing obvious damage to the cytoskeleton, and the addition of GO could alleviate this damage. However, as the dose of MCLR increased, skeletal damage could not be reversed even in the presence of GO. This phenomenon was attributed to the destruction of cell membrane integrity by GO at high dose, which made it easier for MCLR to enter into cells, thus causing more serious damage to the cytoskeleton.

## Conclusion

To sum up, this paper mainly explored the adsorption effect of GO on MCLR, as well as the toxic effect and mechanism of complex GO-MCLR on HaCaT and L02 cells. The adsorption study found that GO could effectively adsorb MCLR through hydrogen bonding and π–π conjugation, the oxygen-containing functional groups of GO-MCLR composite decreased significantly and the disorder degree raised. A series of toxicity experiments suggested that compared to GO or MCLR exposure, GO-MCLR at high dose caused more remarkable apoptosis and ferroptosis in both HaCaT and L02 cells. The underlying mechanism was that GO-MCLR with lower oxidation degree and higher disorder degree induced more intense intracellular ROS and mtROS generation, followed by the accumulation of Fe^2+^, mitochondrial dysfunction and cytoskeletal damage in cells, and eventually leaded to programmed death. The above results revealed the adsorption effect of GO on MCLR in aquatic environment, and clarified the possible toxic effect and mechanism of GO-MCLR complex from the perspective of actual exposure pathway, providing theoretical basis for the ecological risk of graphene materials applied in environmental remediation.

## Methods

### Adsorption of MCLR by GO

MCLR was purchased from ALEXIS® Biochemicals, Switzerland. GO with a thickness of 1.0 nm was kindly provided by the collaborative research group of Zhang Tao at School of Materials Science, Nanjing University, the relevant parameters can be found in zhang's article [59]. A mixed solution of GO-MCLR (50 μg/mL GO + 1 μg/mL MCLR, pH = 7) was prepared by using the GO and MCLR pregnant liquor with a concentration of 1 mg/mL, then place it on the turntable of the hybridization furnace and rotate in dark at 40 r/min, 26 ℃. After 5 min, 10 min, 30 min, 3 h, 12 h and 24 h, the sample was centrifuged (12,000 r/min, 15 min) and 200 μL of supernatant was collected. MCLR concentration in the supernatant was detected by high performance liquid chromatography (HPLC) referring to Yang's method [60]. The concentration reduction was obtained by subtracting the concentration in the supernatant at each time point from the initial concentration, and the adsorption amount of GO to MCLR at the above 8 time points was further determined.

### Physicochemical characterizations of GO and GO-MCLR

5 mg of GO was weighed and added to 1 L of ultrapure water, and sonicated for 30 min to afford evenly dispersed GO suspension. MCLR solution (60 µL), in a concentration of 1 mg/mL, was added to prepared GO suspension, GO-MCLR composite solution was obtained after well mixing. A drop of GO/GO-MCLR solution was placed on the copper net and slowly dried with nitrogen, then morphologies of GO/GO-MCLR were observed and photographed by by spherical aberration corrected Transmission Electron Microscope (ACTEM) (FEI TITAN 80-300 operating at 80 kV). The hydration particle sizes of all samples were obtained by Malvern laser particle size analyzer (ZetasizerNano-ZS90, Malvern Instruments, UK). The surface chemical states and functional groups of GO and GO-MCLR were investigated by FTIR (Nicolet IS20, Thermo Fisher Technology LTD, China) and XPS (Shimadzu, Japan). Microscopical laser Raman spectroscopy (MLRM) (Renishaw inVia Reflex, Wotton under Edge, UK) was used to obtain atomic vibration, phonon scattering and other information to study the molecular structure of GO and GO-MCLR.

### Cell culture

Both HaCaT cells and L02 cells were procured from the Cell Bank of the Chinese Academy of Sciences (Shanghai, China), and were cultured with Dulbecco's modified Eagle's medium (DMEM) added with 10% (v/v) FBS (Biological Industries, Karmiel, Israel), 10kU/mL penicillin, 10 mg/mL streptomycin and 5 mg/mL gentamicin (Solarbio, Beijing, China) as cell medium in a 37 ℃ cell incubator containing 5% CO_2_. According to the adsorption amount of GO to MCLR when adsorption equilibrium, cells for cytotoxicity studies were grouped as follows: Control group, GO groups (5 µg/mL and 50 µg/mL), MCLR groups (0.06 µg/mL and 0.6 µg/mL) and GO-MCLR complex groups (5 µg/mL + 0.06 µg/mL, 50 µg/mL + 0.6 µg/mL).

### Cytotoxicity assessment

Cell viability: Cells at logarithmic growth stage were adjusted to 1 × 10^5^ /mL and inoculated in 96-well plates. After overnight culture, the original medium was abandoned and the medium containing different concentrations of GO, MCLR and GO-MCLR was added for poisoning. After 24 h of exposure, the culture medium was removed and 10% Cell Counting Kit-8 (CCK-8, Beyotime Biotechnology, Shanghai, China) solution was added. In order to prevent the interference of GO on the results, the supernatant was centrifuged (10 min, 14,000 r/min) and transferred to a new 96-well plate before the solution absorbance (OD value) was detected at wavelength 570 nm by the Epoch multi-volume spectrophotometer system (BioTek, USA).

Cell membrane integrity: Cell membrane integrity was assessed using the Lactate dehydrogenase (LDH) cytotoxicity assay kit (Beyotime Biotechnology, Shanghai, China) according to manufacturer's instructions. After 24 h of poisoning, the culture plates were centrifuged at 400 g for 5 min, and 120 µL of supernatant was taken from each well into a new 96-well plate. Reaction away from light for 30 min after addition of LDH working solution was carried out, and then the OD value was detected at wavelength 490 nm.

Cell morphology observation: After incubation for 24 h, the poisoning solution in the wells was discarded, and cells were rinsed by PBS three times. The changes in cell volume, morphological profile and cell number were observed under Inverted microscope (CK40, Olympus Corporation, Japan).

### Flow cytometry

Annexin V-FITC/PI apoptosis kit (BD Biosciences, USA) was applied for detection of apoptosis in treated cells by flow cytometry (FACS Aria II, Becton Dickinson, San Jose, CA). The treated cells were collected by centrifugation after washing. Then harvested cells were gently mixed with 300 μL Annexin V-FITC binding solution and 5 µL Annexin V-FITC, and incubated for 15 min at room temperature, away from light. 5 min before detection, 5 µL PI and 200 μL Annexin V-FITC binding solution were added. FITC (FL1) and PI (FL3) channels were selected, and 10,000 cells were analyzed per sample.

### Detection of caspases 3 and 7 activations by fluorescence microscopy

HaCaT/L02 cells were seeded on 24-well slides with a cell density of 4 × 10^4^ cells/mL. After adherence, cells were treated with GO, MCLR and GO-MCLR of different concentrations for 24 h and washed with PBS. According to operation instructions, the cells were incubated with the prepared FLICA caspase 3/7 substrates for 1 h in the dark. The cells were rinsed 3 times with the buffer solution delivered with the kit, subsequently, were stained with Hoechst 33,342 probe and imaged by fluorescence microscope (Carl Zeiss AG, Oberkochen, Germany).

### Measurement of intracellular Fe^2+^ level

The content of Fe^2+^ in the cells was measured by FerroOrange ferrous ion fluorescence probe (Maokang Bio-Technology Co., LTD, Shanghai, China). HaCaT/L02 cells were pretreated in line with the above, and FerroOrange working solution prepared according to the manufacturer's protocol was added. After incubation for 1 h keeping out light, Cells were observed and imaged using the g-excitation filter in fluorescence microscopy.

### Western blotting

Total proteins of HaCaT/L02 cells were extracted for Western blotting assay, which was completed as described before. Anti-SLC7A11, Anti-GPX4, Anti-β-Tublin, HRP Goat Anti-Rabbit IgG and marker in this paper were supplied by Abclonal (Wuhan, China). Fluorescence signal were detected after incubated in West Pico chemiluminescence reagent (Pierce, Rockford, IL, USA).

### Detection of intracellular ROS and oxidative stress

Intracellular ROS identified by DCFH-DA (Beyotime Biotechnology, Shanghai, China) following the manufacturer’s instructions were captured by fluorescence microscope. Assessment of oxidative stress was determined through intracellular SOD, MDA and GSH levels, which were obtained using SOD, MDA and GSH kits.

### Assessment of mitochondrial injury

Mitochondrial injury was assessed by mtROS, mitochondrial MMP, ATP generation and intracellular Ca^2+^ levels. MitoSOX™ red mitochondrial superoxide dismutase indicator (Thermo Fisher Scientific, USA) was used to detect mtROS levels. The fluorescent probe JC-1 was used to indicate the change of MMP. ATP generation was measured by the ATP assay kit using a microplate luminescence detector (Berthold Technologies, Germany) as per the developer's instructions. HaCaT/L02 cells were stained with Fluo-3AM fluorescence probe (Beyotime Biotechnology, Shanghai, China) to detect the level of Ca^2+^ in the cells. Then cells were observed and imaged using the fluorescence microscopy at excitation wavelength of 506 nm and emission wavelength of 526 nm.

### Detection of PP2A activity by ELISA

After 24 h of treatment, the cells culture medium was collected and analyzed for PP2A levels, which were detected by ELISA kit. Specific steps were completed following the instructions of the manufacturer. Finally, the OD value of the solution was measured at 450 nm with a microplate reader (BIOTEK, USA).

### Observation of cytoskeleton

After 24 h of exposure, the cells were fixed with 4% paraformaldehyde dissolved in PBS for 10 min and washed with PBS twice. Then the cells were treated at room temperature for 5 min with 0.5%Triton X-100 dissolved in PBS, and cleaned with PBS twice. 200 µL FITC-Phallodin staining solution was added and incubated for 30 min at room temperature without light. Eventually, 100 µL anti-fluorescence quenching agent containing DAPI was added, and the staining results were imaged under laser confocal microscopy. The FITC (Ex/Em = 492/518 nm) and DAPI (Ex/Em = 364/454 nm) excitation/emission filters were selected.

### Statistical analysis

The data were expressed as mean ± standard deviation ($\overline{\chi }$ ± SD), and SPSS20.0 was used to perform independent sample T test and one-way ANOVA. Comparisons between the treatment group and the control group were analyzed by Dunnett-t two-tailed test. *P* < 0.05 indicated that the difference between the two groups was statistically significant.

## Supplementary Information

**Additional file 1.** Pyroptosis of HaCaT and L02 cells after treatment by flow cytometry. The immunoblotting bands and relative intensities of GSDMS by western blot. The caspases 1 expression in HaCaT and L02 cells by Confocal microscopy images. The adsorption kinetics model of GO for MCLR. The adsorption isotherm of GO for MCLR. This material is available free of charge via the Internet at https://particleandfibretoxicology.biomedcentral.com/.

## Data Availability

All data and materials are included in the manuscript, figures and Additional file 1.

## Abbreviations

ATP: Adenosine triphosphate
EDTA: Ethylene diamine tetraacetic acid
FTIR: Fourier transform infrared spectrometer
GO: Graphene oxide
GSH: Glutathione
HaCaT cells: Human skin cutin forming cells
HPLC: High performance liquid chromatography
L02 cells: Normal liver cells
LDH: Lactate dehydrogenase
MCs: Microcystins
MCLR: Microcystin-LR
MDA: Malondialdehyde
MLRM: Microscopical laser Raman spectroscopy
MMP: Membrane potential
mtROS: Mitochondrial ROS
PP1: Protein phosphatase 1
PP2A: Protein phosphokinase 2A
ACTEM: Aberration corrected transmission electron microscope
ROS: Reactive oxygen species
SOD: Superoxide dismutase
XPS: X-ray photoelectron spectroscopy
