# Supplementary material for: Safety assessment of graphene oxide and microcystin-LR complex: a toxicological scenario beyond physical mixture
Source: Part Fibre Toxicol. 2022 Apr 7;19:26. doi: 10.1186/s12989-022-00466-x (PMC8988332; doi:10.1186/s12989-022-00466-x)
Supplement: Supplementary file 1 — Additional file 1. Pyroptosis of HaCaT and L02 cells after treatment by flow cytometry. The immunoblotting bands and relative intensities of GSDMS by western blot. The caspases 1 expression in HaCaT and L02 cells by Confocal microscopy images. The adsorption kinetics model of GO for MCLR. The adsorption isotherm of GO for MCLR. This material is available free of charge via the Internet at https://particleandfibretoxicology.biomedcentral.com/. [file 12989_2022_466_MOESM1_ESM.docx]

**Supplementary Methodology**

**Cell pyrophosis by flow cytometry**

Cells pyroptosis level was determined by flow cytometry. HaCaT/L02 Cells at logarithmic growth stage were cultured in 12-well plates with density of 1×10^5^ /mL. After overnight culture, cells were pretreated with LPS (200 ng/mL) for 2 h and then different concentrations of GO, MCLR and GO-MCLR was added. Cells were collected 24 hours later for detection according to the kit instructions.

**Western blotting**

Total proteins of HaCaT/L02 cells were extracted for Western blotting assay, which was completed as described before. Anti-GSDMD, Anti-β-Tublin, HRP Goat Anti-Rabbit IgG and marker in this paper were supplied by Abclonal (Wuhan, China). Fluorescence signal were detected after incubated in West Pico chemiluminescence reagent (Pierce, Rockford, IL, USA).

**Detection of Casepes1 activation by fluorescence microscopy**

HaCaT/L02 cells were seeded on 24-well slides with a cell density of 4×10^4^ cells/mL. After adherence, cells were treated with GO, MCLR and GO-MCLR of different concentrations for 24 h and washed with PBS. According to operation instructions, the cells were incubated with the prepared FLICA caspase-1 substrates for 1 h in the dark. The cells were rinsed 3 times with the buffer solution delivered with the kit, subsequently, were stained with Hoechst 33342 probe and imaged by fluorescence microscope(Carl Zeiss AG, Oberkochen, Germany).

**Assay of adsorption kinetics**

The kinetic studies done with MCLR 1000 μg/L for 24 h in the presence of GO 50 mg when pH = 7, the mixed liquid was placed on the turntable of the hybridization furnace and rotate in dark at 40 r/min, 26 ℃. After 5 min, 10 min, 30 min, 3 h, 12 h and 24 h, the sample was centrifuged (12000 r/min, 15 min) and 200 μL of supernatant was collected. The free MCLR concentration in the supernatant was detected by high performance liquid chromatography (HPLC).

**Assay of adsorption isotherm**

The Adsorption isotherm done with MCLR (100, 200, 400, 600, 800 and 1000 μg/L) for 24 h in the presence of GO 50 mg when pH = 7. the mixed liquid was placed on the turntable of the hybridization furnace and rotate in dark at 40 r/min, 26 ℃. After 24 h, the sample was centrifuged (12000 r/min, 15 min) and 200 μL of supernatant was collected. The free MCLR concentration in the supernatant was detected by high performance liquid chromatography (HPLC). Then Langmuir model was used to describe the non-linearequilibrium relationship between the solute sorbed onto the sorbent and that left in solution. The model equations can be represented as,

Langmuir model :$Q_{e}=\frac{Q_{m}K_{L}C_{e}}{1+K_{L}C_{e}}$

where Qm is the maximum MCLR uptake, K_L_ is the Langmuir equilibrium constant and Ce is equilibrium concentration.

**Supplementary Figure 1**

**
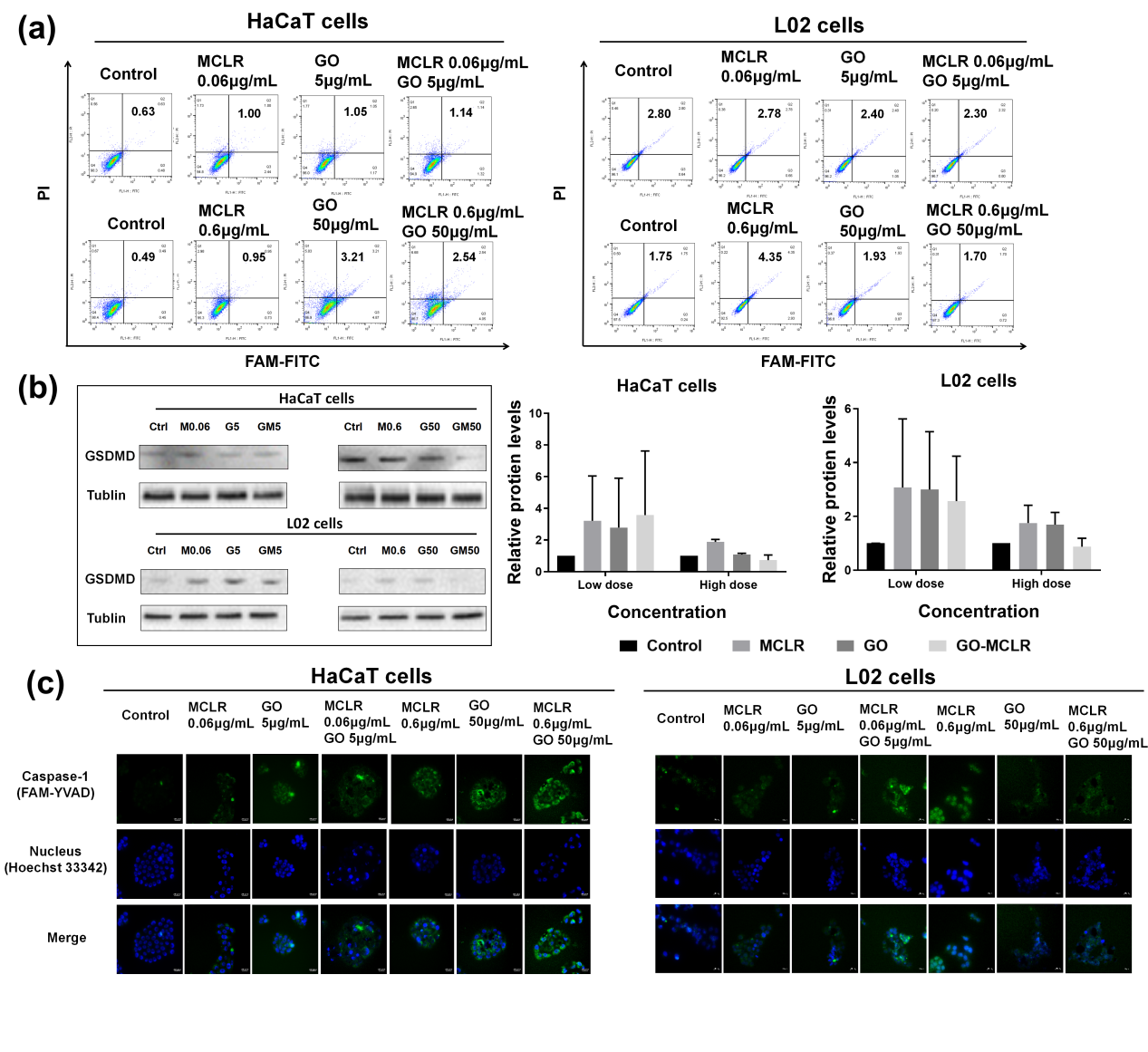
Figure S1. Effects of GO, MCLR and GO-MCLR on Pyroptosis in HaCaT and L02 cells.** HaCaT and L02 cells were pretreated with LPS (200 ng/mL) for 2 h and then different concentrations of GO, MCLR and GO-MCLR was added. (a)Cells were collected 24 hours later for detection of pyroptosis by flow cytometry. (b)The immunoblotting bands and relative intensities of GSDMS in treated cells were shown. Band intensities were quantified and normalized to Tublin. **P*<0.05 vs. Control, #*P*<0.05 vs. GO group. (c)Confocal microscopy images showing GO, MCLR and GO-MCLR-induced caspases 1 expression in HaCaT and L02 cells. After 24 h of exposure, Cells were incubated with the prepared FLICA caspase 3/7 substrates for 1 h in dark and rinsed with the buffer solution. Cell nuclei were stained with Hoechst 33342.

**Supplementary Figure 2**

**
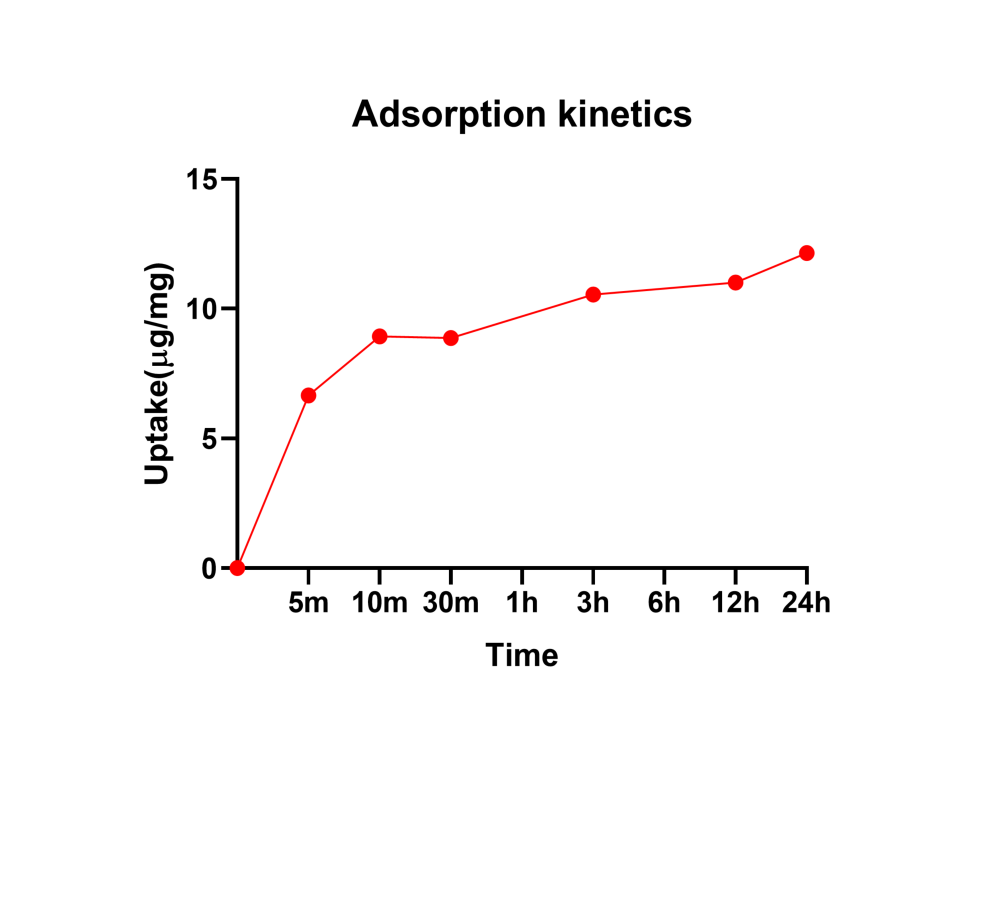
Figure S2. The Adsorption kinetics model of GO for MCLR.** The kinetic studies done with MCLR 1000 μg/L for 24 h in the presence of GO 50 mg when pH = 7.

**Supplementary Figure 3**

**Figure S3. The Adsorption isotherm of GO for MCLR.** The Adsorption isotherm done with MCLR (100, 200, 400, 600, 800 and 1000 μg/L) for 24 h in the presence of GO 50 mg when pH = 7. Langmuir model was used to describe the non-linearequilibrium relationship between the solute sorbed onto the sorbent and that left in solution. The model equations can be represented as,

Langmuir model :$Q_{e}=\frac{Q_{m}K_{L}C_{e}}{1+K_{L}C_{e}}$

where Q_e_ is the adsorption capacity at equilibrium, Q_m_ is the maximum MCLR uptake, K_L_ is the Langmuir equilibrium constant and C_e_ is equilibrium concentration.

.
